# Ecological traits affect the sensitivity of bees to land‐use pressures in European agricultural landscapes

**DOI:** 10.1111/1365-2664.12524

**Published:** 2015-09-23

**Authors:** Adriana De Palma, Michael Kuhlmann, Stuart P.M. Roberts, Simon G. Potts, Luca Börger, Lawrence N. Hudson, Igor Lysenko, Tim Newbold, Andy Purvis

**Affiliations:** ^1^ Department of Life Sciences Imperial College London Silwood Park Berkshire SL5 7PY UK; ^2^ Department of Life Sciences Natural History Museum Cromwell Road London SW7 5BD UK; ^3^ Centre for Agri‐Environmental Research School of Agriculture, Policy and Development The University of Reading Reading RG6 6AR UK; ^4^ Department of Biosciences College of Science Swansea University Singleton Park Swansea SA2 8PP UK; ^5^ United Nations Environment Programme World Conservation Monitoring Centre 219 Huntington Road Cambridge CB3 0DL UK

**Keywords:** biodiversity, ecosystem services, human impacts, land‐use change, land‐use intensification, life‐history traits, pollinators

## Abstract

1. Bees are a functionally important and economically valuable group, but are threatened by land‐use conversion and intensification. Such pressures are not expected to affect all species identically; rather, they are likely to be mediated by the species' ecological traits.
2. Understanding which types of species are most vulnerable under which land uses is an important step towards effective conservation planning.
3. We collated occurrence and abundance data for 257 bee species at 1584 European sites from surveys reported in 30 published papers (70 056 records) and combined them with species‐level ecological trait data. We used mixed‐effects models to assess the importance of land use (land‐use class, agricultural use‐intensity and a remotely‐sensed measure of vegetation), traits and trait × land‐use interactions, in explaining species occurrence and abundance.
4. Species' sensitivity to land use was most strongly influenced by flight season duration and foraging range, but also by niche breadth, reproductive strategy and phenology, with effects that differed among cropland, pastoral and urban habitats.
5. *Synthesis and applications*. Rather than targeting particular species or settings, conservation actions may be more effective if focused on mitigating situations where species' traits strongly and negatively interact with land‐use pressures. We find evidence that low‐intensity agriculture can maintain relatively diverse bee communities; in more intensive settings, added floral resources may be beneficial, but will require careful placement with respect to foraging ranges of smaller bee species. Protection of semi‐natural habitats is essential, however; in particular, conversion to urban environments could have severe effects on bee diversity and pollination services. Our results highlight the importance of exploring how ecological traits mediate species responses to human impacts, but further research is needed to enhance the predictive ability of such analyses.

## Abstract 2

Rather than targeting particular species or settings, conservation actions may be more effective if focused on mitigating situations where species' traits strongly and negatively interact with land‐use pressures. We find evidence that low‐intensity agriculture can maintain relatively diverse bee communities; in more intensive settings, added floral resources may be beneficial, but will require careful placement with respect to foraging ranges of smaller bee species. Protection of semi‐natural habitats is essential, however; in particular, conversion to urban environments could have severe effects on bee diversity and pollination services. Our results highlight the importance of exploring how ecological traits mediate species responses to human impacts, but further research is needed to enhance the predictive ability of such analyses.

## Introduction

Bees are key providers of pollination services, which are vital for food security and the persistence of many wild plants (Klein *et al*. 2007; Ollerton, Winfree & Tarrant 2011). However, many bee species are threatened by changing and intensifying land use (Potts *et al*. 2010; Ollerton *et al*. 2014).

Land‐use change, such as conversion from semi‐natural habitats to human‐dominated land uses, can greatly impact bee communities. Urbanization, agricultural expansion and abandonment are ongoing drivers of land‐use change in Europe (Verburg *et al*. 2006), which can affect bee diversity through reduced floral and nesting resources (Hernandez, Frankie & Thorp 2009; Forrest *et al*. 2015). Semi‐natural habitats are prime targets for land conversion (Verburg *et al*. 2006). Such habitat loss can affect pollination of crops as well as of wild flowers: as central place foragers, bees often forage up to a few kilometres away from their nests (Greenleaf *et al*. 2007) so semi‐natural habitat can provide spillover of pollination services to nearby cropland and vice versa (Blitzer *et al*. 2012).

Agricultural intensification – through decreased crop diversity and increased external inputs – is another major pressure, which can impact bees directly by increasing mortality and indirectly by decreasing resource availability (Potts *et al*. 2010; Roulston & Goodell 2011). For instance, neonicotinoid pesticides restrict colony growth and queen production in bumblebees and limit foraging success and survival of honeybees (Henry *et al*. 2012; Whitehorn *et al*. 2012). Nitrogen fertilizer and herbicides can affect bees indirectly by reducing the diversity of plants (Kleijn *et al*. 2009) and thus foraging resources (Roulston & Goodell 2011). Reductions in non‐crop habitat as management intensifies can reduce the availability of nesting sites, while increased tillage in cropland disturbs the nesting sites of some species (Shuler, Roulston & Farris 2005).

These pressures are unlikely to affect all species identically, but are expected to be mediated by species' traits (Murray, Kuhlmann & Potts 2009; Roulston & Goodell 2011). In general, species with narrower niches – in terms of space, time, phenotype, or interspecific interactions – are predicted to be more sensitive than generalists (Den Boer 1968; Kassen 2002). Bee species' traits may specifically influence vulnerability to land use; for instance, larger foraging ranges facilitate foraging in fragmented landscapes, but may increase the likelihood of contact with pesticides and indicate greater resource needs. Other traits can influence susceptibility to demographic stresses and stochastic events; for example, a higher reproductive capacity may buffer species against disturbances, but may indicate greater resource requirements.

Identifying traits that render species vulnerable to human impacts can help inform and guide effective conservation priorities. Most previous attempts to identify ecological correlates of bee vulnerability to human impacts have focused on a relatively small number of sites and threats, or on museum collections rather than ecological survey data (e.g. Vázquez & Simberloff 2002; Bartomeus *et al*. 2013a). One exception is Williams *et al*.'s (2010) global multi‐species analysis, which found that some traits correlated with vulnerability to multiple threats: for instance, above‐ground versus below‐ground nesting influenced species' susceptibility to fire, isolation and agricultural management practices. Vulnerability traits can also be threat‐specific (Owens & Bennett 2000; Purvis *et al*. 2005), in which case conservation actions would need to focus on populations experiencing ‘dangerous’ combinations of local pressures and ecological traits. For instance, social species may be more sensitive in intensively used cropland – where enhanced foraging capacity can increase exposure to pesticides and thus affect mortality and colony success – but relatively less sensitive in urban areas, where greater foraging capacities may enable persistence (Banaszak‐Cibicka & Żmihorski 2011).

In the broadest analysis of European bees to date, we explore whether ecological traits influence the responses of 257 bee species to local land‐use pressures at 1584 European sites. Unlike the study by Williams *et al*. (2010), we analyse multiple traits within the same models. We aim to identify the traits and land‐use pressures associated with a species having low probability of occurrence and low abundance; we also aim to estimate the relative importance of land use, traits and the interaction between them in shaping species' occurrence and abundance. We hypothesize that resource and phenological niche breadth, foraging range and reproductive strategy will all influence species' sensitivity to land use.

## Materials and methods

### Data collation

Data were sought from published comparisons where bee abundance and occurrence were sampled in multiple sites within agricultural landscapes. Papers based on potentially suitable data were identified by systematically searching Web of Science during 2011–2012 (Table S1.1, Supporting Information), searching journal alerts and assessing references cited in reviews. Criteria for selection were as follows: (i) multiple European sites were sampled for bee abundance or occurrence using the same sampling method within the same season; (ii) at least one site was <1 km from agricultural land; (iii) geographic coordinates were available for each site and (iv) sites were sampled since February 2000, so that diversity data could be matched with remote‐sensed data from NASA's Moderate Resolution Imaging Spectroradiometer (MODIS). MODIS data were chosen over other remote‐sensed imagery as they are available at high spatial (250 m) and temporal (16 days) resolutions and are easily integrated into R analyses (Tuck *et al*. 2014).

We extracted site‐level occurrence and abundance data from suitable papers where possible. Raw data were usually not included within the paper or supplementary files so we asked corresponding authors for these data. Relevant data were available from 30 papers, hereafter referred to as sources (Table S1.2). Some sources separately report data collected in different ways or seasons. We term each separate data set a ‘study’: within, but not between, studies, diversity data can be compared straightforwardly among sites because sampling protocols were the same. We also split data sets that spanned multiple countries into separate studies for each country to account for biogeographic variation in diversity. Differences in sampling effort within a study were corrected for, assuming that recorded abundance increases linearly with sampling effort. Within each study, we recorded any blocked or split‐plot design. In all but one case, this was the sampling design of an observational study. Only one study included was an experimental project, where only the control data were extracted; this study had extremely low influence on the final models (based on Cook's distance, influence.ME package, Niewenhuis, te Grotenhuis & Pelzer 2012) and did not qualitatively change the results.

The major land use and use intensity at each site was assessed based on information in the associated paper, using the scheme described in Hudson, Newbold *et al*. (2014), reproduced in Table S1.3. Land use was classified as secondary vegetation, cropland, pasture or urban. The use‐intensity scale – a qualitative measure of the extent of human disturbance – is coarse (three levels: minimal, light and intense), but can be applied in a wide range of settings (Hudson, Newbold *et al*. 2014). Many combinations of land use and use intensity had too few sites to permit robust modelling; the data were therefore coarsened into a single factor (hereafter, Land Use and Intensity, LUI), collapsing levels to ensure adequate sample sizes. The final data set had the following LUI classes: secondary vegetation (165 sites), minimally‐used cropland (168), lightly‐used cropland (415), intensively‐used cropland (653), pasture (138) and urban (45).

As well as using a coarse, discrete representation of land use, we also used remotely sensed mean Normalized Difference Vegetation Index (NDVI), to capture additional variation in vegetation between sites. NDVI is highly correlated with above‐ground biomass and net primary productivity (Pettorelli *et al*. 2005) and often correlates positively with plant and invertebrate species richness even at relatively small spatial scales (e.g. Gould 2000; Lassau & Hochuli 2008). For each site, we downloaded MODIS MOD13Q1 (collection 5) NDVI data (composited for 16 days) at 250‐m spatial resolution for up to 3 years, with the final year being the year of sampling. Poor‐quality observations were removed and linear interpolation applied to remaining data. The time series was averaged to give mean NDVI (henceforth, mNDVI). NDVI data were downloaded and processed using the MODISTools package (Tuck *et al*. 2014). In our data set, high mNDVI is unlikely to be driven by densely forested areas (which may not benefit bees in temperate systems, Winfree *et al*. 2007): wooded sites were only present in two of 24 sources (three sites in woodland and two in mixed woodland and agriculture) and these sources were not particularly influential in the final models (as judged by Cook's distance values; all ≤0·097).

Data on species traits were compiled by SR and MK; morphometric data came from museum specimens and other traits from many published and unpublished sources (Table S2.1). We used traits reflecting resource specialization, phenology, reproductive strategy and foraging range. Flight season duration and intertegular distance were treated as continuous variables, and all other traits as factors. Sample sizes were increased by collapsing factor levels where necessary to permit robust modelling (Table 1 and Table S2.1).

**Table 1:** Ecological traits and categories (after coarsening) available for European bee species. Numbers in parentheses indicate the number of species with these traits

Trait of interest	Proxy for trait of interest	Explanation
Niche Breadth	Lecty Status: Obligately oligolectic (63) Polylectic/Flexible (147) Species with no lecty status (47)	Obligately oligolectic species can be monolectic (foraging on one plant species) or oligolectic (forage on plants from less than four genera). Polylectic species are generalist foragers (collecting pollen from five or more plant genera) (Murray, Kuhlmann & Potts 2009). Species that can be polylectic are placed within the latter group. Species with no lecty status are parasitic (they lay eggs in other species' nests) so do not collect pollen, but may respond more quickly to disturbance than other species, thus indicating the status of the total bee community (Sheffield *et al*. 2013).
	Tongue Length: Short (157) Long (100)	This is a family‐specific trait, not the physical tongue length of each individual or species. It has been suggested that long‐tongued bumblebees tend to forage on Fabaceae, and so are more specialized than short‐tongued species (Goulson *et al*. 2005).
	Nesting Strategy: Excavators (141) Pre‐existing cavity dwellers (116)	Excavators are species that excavate their own nests, often requiring bare hard ground or pithy stems; in this analysis, all species in this category nest below‐ground, but one. Pre‐existing cavity dwellers (e.g. bumblebees) nest above‐ground in pre‐existing cavities such as empty snail shells, regardless of nest location, or are parasitic (Potts *et al*. 2005).
Phenology	Duration of the flight season: From 2 to 12 months (257)	Longer flight seasons increase the number of flowering species with which a bee overlaps. Flight season duration is calculated using the earliest and latest date in the year a specimen has ever been recorded; in reality, this is an overestimate as phenology depends on weather conditions that vary between years.
	Voltinism: Obligately univoltine (224) Multivoltine/Flexible (33)	Multivoltine species lay eggs multiple times throughout the year (most are bivoltine, laying twice), and so have a higher reproductive capacity than univoltine species which lay only one brood per year. Univoltine species may be particularly vulnerable to disturbances that coincide with the time of reproduction (Brittain & Potts 2011). Voltinism can vary with geography and the climate; species that can vary brood production depending on environmental conditions are classed as multivoltine/flexible.
Reproductive strategy	Sociality: Obligately solitary (203) Not obligately solitary (54)	Social bees have a higher foraging and reproductive capacity, and have a faster response to resource provision, than solitary bees, which may buffer them against human impacts. However, sociality requires continuous brood production, which may increase time stress and resource requirements. Enhanced foraging capacity may also increase pesticide exposure (as foragers using various resources in different areas may bring pesticide‐containing pollen and nectar back to the nest, Brittain & Potts 2011). Social species also tend to have low effective population sizes, which may make populations more susceptible to human impacts (Chapman & Bourke 2001).
Foraging distance	Intertegular distance (ITD): From one to six mm (257)	ITD is a proxy for dry weight (Cane 1987; Hagen & Dupont 2013) and foraging distance in bees (Greenleaf *et al*. 2007). Although alternative measures of body size do exist (e.g. wingspan), their relationship with foraging distance is either understudied or inconsistent among genera (Cane 1987; Westphal, Steffan‐Dewenter & Tscharntke 2006). Only data for females were used.

### Analysis

We excluded 14 sites for which LUI or mNDVI was not available, and 12 species for which not all trait values were known.

The diversity data were zero‐inflated with a positive mean–variance relationship, but were not exclusively counts (because abundance measurements included densities) so a discrete error distribution (e.g. Poisson) could not be used. Instead, the analysis was carried out in two stages, equivalent to a hurdle model, using mixed‐effects models (lme4 package version 1.1‐6, Bates, Maechler & Bolker 2013). Species presence (and detection) was modelled using a binomial error structure; then, the (log‐transformed) abundance of present species was modelled using normal errors (Newbold *et al*. 2014). Model assumptions were checked and found to be reasonable (e.g. Fig. S3.1).

We used mixed‐effects models to account for non‐independence of data due to differences in collectors (source), sampling methodologies and biogeography (study), the spatial structure of sites (block), and taxonomy (family and species). The initial, maximal random‐effects structure was block (nested within study within source), crossed with species (nested within family). We also tested an alternative structure of block (nested within study within sampling method), but this performed less well (results not shown), so was not pursued. More complicated random‐effect structures (e.g. random slopes) could not be fitted due to computational limitations. Both the presence and abundance models had the same initial maximal fixed‐effects model structure, containing all land‐use (LUI and mNDVI) and trait variables, as well as all two‐way interactions between land use and traits. We determined the best random‐effects structures using likelihood ratio tests (Zuur *et al*. 2009), comparing all formulations.

Full models were assessed for multicollinearity using generalized variance inflation factors (GVIFs, Zuur *et al*. 2009), which never breached the threshold of 10 (Table S3.1 and S3.2). We used backwards stepwise model simplification based on likelihood ratio tests to reduce model complexity as far as possible and to determine whether interactive effects between traits and land use were retained in the final model (Zuur *et al*. 2009). Model simplification reduced the GVIFs (Table S3.3). We assessed robustness of parameter estimates by bootstrapping data points, using 1000 iterations for the abundance model and (because of computational limitations) 100 iterations of the occurrence model. We inferred significance of parameter estimates from the 95% bootstrapped confidence intervals (bCIs, Canty & Ripley 2014) and computed anova tables using type III Wald tests (car package, Fox & Weisberg 2011).

Where the minimum adequate model included significant trait × land‐use interactions, we evaluated the relative importance of land use, traits and their interactions. The following models were constructed for both species occurrence and abundance (if present):

1. Interactive model: the minimum adequate model
2. Additive model: as 1, but with all interactions removed
3. Traits model: as 2, but with all land‐use variables removed
4. Land‐use model: as 2, but with all trait variables removed
5. Null model: only random effects included.

The importance of interactive terms was assessed by comparing the additive model with the interactive model; the importance of traits versus land use was assessed by comparison with the additive model. We chose not to use information criteria for these comparisons. Akaike's information criterion, with its low penalty per extra parameter (2 units), can overestimate the importance of predictors with more parameters when, as here, the data set is large (Link & Barker 2006; Arnold 2010), whilst the penalty for the Bayesian information criterion (the log of the sample size) can be too stringent when, as here, the data are not independent (Jones 2011). Calculating appropriate penalty terms for complex mixed‐effects models is far from straightforward (Delattre, Lavielle & Poursat 2014). We therefore assessed the relative importance of interactive effects in the minimum adequate models using marginal R^2^_GLMM_ values (R^2^ for mixed models), that is the variance explained by fixed effects alone (Barton 2013; Nakagawa & Schielzeth 2013). Specifically, we calculated the decrease in explanatory power when the predictor set of interest was excluded from the model (similar to the process for linear models in Ray‐Mukherjee *et al*. 2014), as a percentage of the marginal R^2^_GLMM_ when the predictor set was included. We used the same approach to estimate the importance of each trait and each land‐use variable separately. These ‘unique’ contributions of focal predictors when isolated from other variables may underestimate or overestimate the full contribution of the focal predictors, depending on the covariation among explanatory variables.

We performed a randomization test to ensure that differences in R^2^_GLMM_ values were not merely caused by differences in model complexity (Nakagawa & Schielzeth 2013). In each trial (1000 for abundance models and 100 for occurrence models), we randomized the species names in the trait data set, conserving the between‐trait correlations and data set structure, but breaking any link between traits and occurrence or abundance. We calculated marginal R^2^_GLMM_ values from interactive, additive and traits‐only models fitted to the randomized data (the land‐use‐only and null models were unaffected by the randomization). We counted how often marginal R^2^_GLMM_ from the randomized data exceeded that of the original models, and expressed the difference as a z‐score. If interactive models are favoured simply because they have more parameters (i.e. a bias caused by an incorrect penalty for complexity), the observed marginal R^2^_GLMM_ will be approximately the average of the values across randomizations.

All analyses were carried out using R: A Language and Environment for Statistical Computing version 2.15.3 (R Core Team 2013).

## Results

### Model results

Many trait × land‐use interactions were retained after model simplification, explaining a significant amount of variation in both species occurrence and abundance if present (Tables 2 and 3; see Table S4.1 and S4.2 for full coefficients). Effects of trait × land‐use interactions were often different for species occurrence and abundance. A decrease in the number of species might enable remaining species to persist at higher abundances (Newbold *et al*. 2014).

**Table 2:** anova table for minimum adequate model of probability of presence

Term	χ^2^	d.f.	Sig
(Intercept)	52·19	1	***
LUI	64·71	5	***
mNDVI	28·39	1	***
Sociality	4·18	1	*
Lecty status	32·11	2	***
Tongue length guild	2·53	1	
Voltinism	0·32	1	
Duration of flight season	18·32	1	***
ITD	5·75	1	*
Nest construction	0·00	1	
LUI × Sociality	36·20	5	***
mNDVI × Sociality	16·90	1	***
LUI × Lecty status	66·39	10	***
mNDVI × Lecty status	31·20	2	***
LUI × Tongue length guild	11·33	5	*
mNDVI × Tongue length guild	7·75	1	**
LUI × Voltinism	48·66	5	***
LUI × Duration of flight season	43·81	5	***
mNDVI × Duration of flight season	5·30	1	*
LUI × ITD	45·15	5	***
mNDVI × ITD	12·18	1	***
LUI × Nest construction	25·23	5	***

Stars indicate the level of significance (Sig): <0·05*, <0·01** and <0·001***. The minimum adequate model had a marginal R^2^_GLMM_ of 0·07 and a conditional R^2^_GLMM_ of 0·58. LUI, Land use and intensity; ITD, intertegular distance (body size); mNDVI, mean NDVI.

**Table 3:** anova table for minimum adequate model of abundance

Term	χ^2^	d.f.	Sig
(Intercept)	0·37	1	
LUI	12·39	5	*
mNDVI	7·56	1	**
Sociality	4·36	1	*
Lecty status	7·92	2	*
Tongue length guild	11·45	1	***
Voltinism	1·37	1	
Duration of flight season	5·05	1	*
ITD	7·34	1	**
LUI × Sociality	23·76	5	***
mNDVI × Lecty status	9·13	2	*
LUI × Tongue length guild	12·16	5	*
mNDVI × Tongue length guild	21·55	1	***
LUI × Voltinism	40·02	5	***
LUI × Duration of flight season	17·14	5	**
mNDVI × ITD	12·35	1	***

Stars indicate the level of significance (Sig): <0·05*, <0·01** and <0·001***. The minimum adequate model had a marginal R^2^_GLMM_ of 0·02 and a conditional R^2^_GLMM_ of 0·71. LUI, Land use and intensity, ITD, intertegular distance (body size), mNDVI, mean NDVI.

### Importance of trait × pressure interactions

Models where interactions were excluded (additive models) explained 13% and 37% less variation in occurrence and abundance, respectively, than the interactive models did (marginal R^2^_GLMM_, Table 4). Traits were relatively more important than land use: the traits‐only model explained 85% and 70% as much variation in occurrence and abundance, respectively, as the additive model, while land‐use‐only models only explained 9% and 17% as much variation in occurrence and abundance as the additive model (marginal R^2^_GLMM__,_ Table 4). These results are not an artefact of model complexity. The observed occurrence models had higher marginal R^2^_GLMM_ than every randomization (z scores: trait‐only model = 19·87; additive – traits and land use – model = 19·77; interactive model = 15·53). The observed abundance models outperformed every randomization for the interactive model (*z* = 4·69), and 97% of the additive (*z* = 4·49) and trait‐only models (*z* = 5·09).

**Table 4:** The fit to data of a null model, models with traits only and land use only, and additive and interactive models with both land use and traits. The interactive model is the minimum adequate model. AIC may favour more complex models (Link & Barker 2006; Arnold 2010), but AIC weights are presented for comparison. Variance of taxonomic random effects are also given (species within family and family)

Response	Model name	Marginal R^2^_GLMM_	Conditional R^2^_GLMM_	AIC weights	Species within family variance	Family variance
Probability of presence	Null model	0·000	0·552	0·000	1·097	0·131
	Land use only	0·008	0·571	0·000	1·100	0·132
	Trait only	0·053	0·560	0·000	0·803	0·164
	Additive	0·058	0·577	0·000	0·805	0·166
	Interactive	0·067	0·579	1·000	0·830	0·162
Abundance of present species	Null model	0·000	0·692	0·000	0·116	0·018
	Land use only	0·004	0·694	0·000	0·116	0·019
	Trait only	0·010	0·696	0·000	0·102	0·033
	Additive	0·012	0·697	0·000	0·102	0·034
	Interactive	0·020	0·708	1·000	0·104	0·043

Including traits increased models' marginal R^2^_GLMM_ (variance explained by fixed effects), but the conditional R^2^_GLMM_ values (variance explained by fixed and random effects) change less, because the effect of traits can also be explained as taxonomic differences in the random‐effects structure (Table 4, Table S4.3).

### Importance of variables

**Figure 1:**
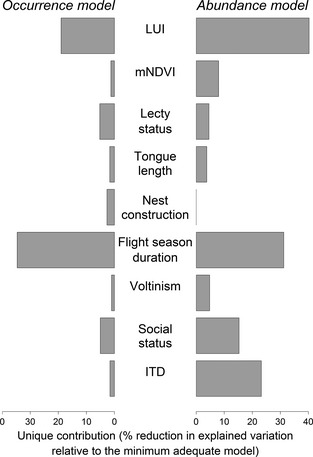
Unique contribution of variables to the explanatory power of minimum adequate models of occurrence and abundance. Contribution is reported as the reduction in variance explained by fixed effects (marginal R^2^ _GLMM_) when the variable and all its interactions are removed from the model, as a percentage of the total variation explained by fixed effects in the minimum adequate models. LUI, Land use and intensity, ITD, intertegular distance (body size), mNDVI, mean NDVI.

Interactions between LUI and traits were more important than interactions between mNDVI and traits (Fig. 1); we therefore focus on the former in the main text (see Appendix S4.1 for full mNDVI results).

**Figure 2:**
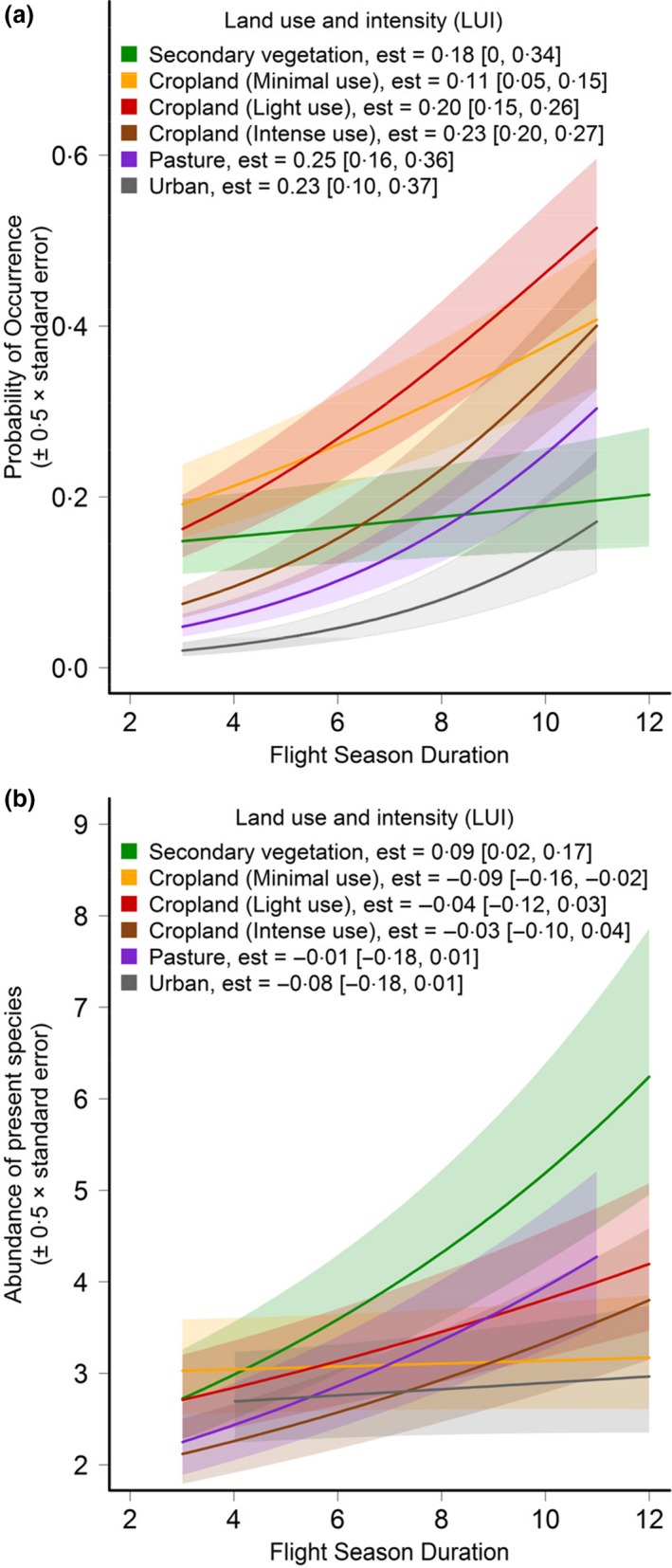
Relationship between flight season duration and a) probability of species presence and b) abundance of present species, in different land uses, as estimated from the minimum adequate models. Error bars represent half the standard error, to ease comparison. The legend indicates the coefficient estimate (est) extracted from the model with 95% bootstrapped confidence intervals (bCIs) in parentheses. The coefficients for human‐dominated land uses are the difference in slope between the given land use and that of secondary vegetation. If bCIs do not cross zero, the estimate is taken to be significant.

In human‐dominated land uses, species with shorter flight seasons were associated with lower probabilities of occurrence than species with longer flight seasons, although the magnitude of the relationship varied among land uses (Fig. 2). Among species that were present, shorter flight seasons were associated with lower abundances in all land uses except for minimally‐used cropland (Fig. 2).

**Figure 3:**
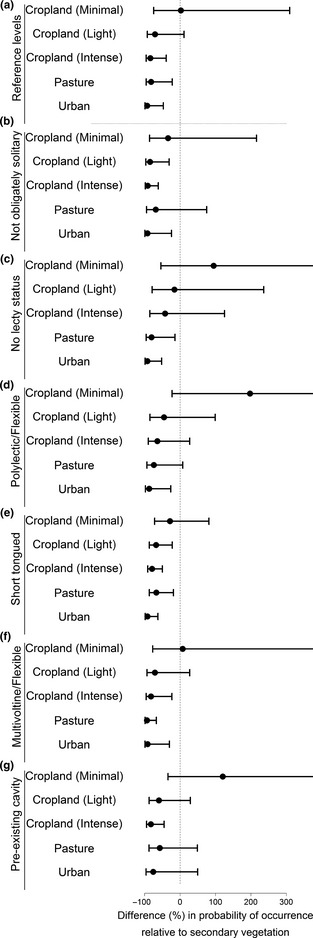
Land use and intensity (LUI) impact on probability of occurrence for species with differing (categorical) ecological traits. For each trait level, this is shown as the % difference in probability of occurrence relative to secondary vegetation, with 95% confidence intervals (CIs) calculated from the model. The trait reference levels in the models were obligately oligolectic, solitary, univoltine, long‐tongued and nest‐excavating species. The effect of LUI on species with these trait values is presented in panel a, and the effects on species with other trait values in panels b–g. Therefore, to compare the sensitivity of long‐tongued species and short‐tongued species to LUI, one would compare panels a and e. CIs in some panels extend beyond the plot region.

**Figure 4:**
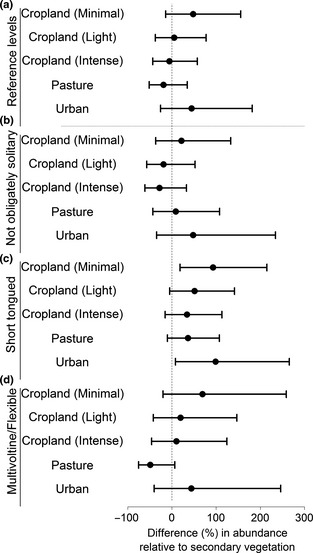
Land use and intensity (LUI) impact on abundance of present species with differing (categorical) ecological traits. For each trait level, this is shown as the % difference in abundance relative to secondary vegetation, with 95% confidence intervals calculated from the model. The trait reference levels in the model included obligately oligolectic, solitary, univoltine and long‐tongued species. The effect of LUI on species with these trait values is presented in panel a, and the effects of species with other trait values in panels b–d. Therefore, to compare the sensitivity of long‐tongued species and short‐tongued species to LUI, one would compare panels a and c.

Other traits were less important in determining species' occurrence and abundance (Fig. 1), but still had significant effects on species sensitivity (Tables 2 and 3). Species with smaller ITD were particularly sensitive to intensively‐used cropland (estimate = 0·11, bCIs: 0·02, 0·18). Oligolectic, solitary, univoltine, long‐tongued and nest‐excavating species were less likely to be present in human‐dominated land uses relative to secondary vegetation, particularly in intensively‐used cropland and urban areas (Fig. 3). If present, however, the abundances of these species did not differ strongly from secondary vegetation (Fig. 4a).

Species with narrower dietary breadths (obligately oligolectic) were generally more sensitive to land use than dietary generalists (Fig. 3a, c, d). Short‐tongued species were sensitive to some land uses in terms of probability of presence (Fig. 3e) but, if present, increased in abundance in some cases (Fig. 4c).

The effects of ecological traits on species' sensitivity were not always consistent across land uses. For example, species that were not obligately solitary were more sensitive than solitary species to lightly‐used cropland (Fig. 3b), but less sensitive to pasture.

## Discussion

Land‐use change and intensification are considered to be major pressures on European bees (Potts *et al*. 2010; Ollerton *et al*. 2014). However, our analyses of 257 species at 1584 sites suggest that these pressures alone explain little of the variation in the presence and abundance of bee species, as effects are often indirect (through reduced floral and nesting resources) and are masked by heterogeneity in species' responses (Roulston & Goodell 2011). We show that species' functional traits – phenology, foraging range, niche breadth and reproductive strategy (sociality) – influence their sensitivity to human‐dominated land use, but do so in ways that differ among cropland, pastoral and urban habitats.

### Land‐use effects on species persistence and abundance

The probability of presence for most species was strongly reduced in intensively‐used cropland relative to secondary vegetation, except for pollen generalists (polylectic, flexible or parasitic species); maintaining stable nesting habitats as well as floral resources may therefore help conserve diversity in such systems (Forrest *et al*. 2015). Species with shorter flight seasons – the most important trait in explaining occurrence and abundance patterns (Fig. 1) – were less likely to be present and were less abundant in intensively‐used cropland, perhaps as this trait confers a higher risk of asynchrony with key floral resources. These results are consistent with previous findings in butterflies that floral specialists with shorter flight seasons are more likely to be rare and threatened (Dennis *et al*. 2004; Barbaro & Van Halder 2009). Previous studies of bees show less consistent patterns, although they assessed relatively few sites and species (e.g. Vázquez & Simberloff 2002; Connop *et al*. 2010). Although our analyses are based on different data sets, these results are similar to those in Williams *et al*. (2010), who found that social species and pollen specialists were particularly sensitive to agricultural intensification.

Small species were also particularly sensitive to intensive agriculture, perhaps because larger species are able to forage further from their nest (Greenleaf *et al*. 2007; Wright, Roberts & Collins 2015). These results suggest that the placement of floral margins will need careful planning with respect to species' nesting habitats (Wright, Roberts & Collins 2015). Long‐distance foraging may increase susceptibility to some landscape‐scale threats (e.g. pesticide exposure), but local conditions are likely to be more important for bee diversity and pollination services in temperate systems (Kennedy *et al*. 2013; Benjamin, Reilly & Winfree 2014).

Even in lightly‐used cropland, short‐tongued species that are not obligately solitary had significantly lower probability of occurrence relative to secondary vegetation, perhaps because their greater foraging breadth and capacity exposes them more to pesticides (Williams *et al*. 2010). In contrast, minimally‐used cropland maintained relatively diverse bee communities – although species with shorter flight seasons were still vulnerable – suggesting an advantage of organic and other low‐intensity farming practices.

Many species were sensitive to pasture, though sociality, polylecty, cavity nesting and long flight seasons were associated with lower sensitivity. Social and polylectic species have enhanced foraging capacity, enabling effective exploitation of available resources and persistence in a patchy mosaic. Small species were also less sensitive to pasture than to other land uses, perhaps because forage is available within smaller distances of nesting sites.

Most species, including those with shorter flight seasons, were less likely to be present in urban areas than in secondary vegetation; only cavity‐nesting species were unaffected. If present, however, most species tended to be fairly abundant, especially short‐tongued species. Our results are congruent with previous studies that have found a negative impact of urbanization on bees (Hernandez, Frankie & Thorp 2009) accompanied by an increase in the numbers of cavity‐nesting species (Hernandez, Frankie & Thorp 2009; Fortel *et al*. 2014). Although other studies have found little difference in diversity between urban areas and semi‐natural habitats (Baldock *et al*. 2015), our results suggest that further loss of secondary vegetation as a result of urbanization may be particularly detrimental to bee communities and to pollination services, as the loss of dietary generalists can greatly affect plant‐pollinator networks (Memmott, Waser & Price 2004).

### Limitations of the study

Our data set is large, but only contains 12·5% of European bee species, with biases towards Western Europe and bumblebees. In addition, little of the variation in species' diversity was explained by fixed effects in our models: most was attributed to heterogeneity between sources (Table S4.3), reflecting differences in sampling methodology, intensity and timing, as well as land‐use practices or pressures that we did not consider. In addition, we used a small number of species' functional traits that were coarsely categorized and omitted intraspecific variation. Further collation of relevant trait information could greatly enhance the predictive ability of models such as these.

Some effects may be influenced by differential detectability; for instance, larger species that are active for longer are more likely to be sampled. This is in part why we have focussed on differences in sensitivity – changes between secondary vegetation and human‐dominated land uses – rather than absolute differences in occurrence and abundance between species. However, detectability may vary among land uses. For instance, with visual sampling methods such as aerial transects, small species may be less frequently sampled in denser vegetation where they are more difficult to see. This may be in part accounted for by the inclusion of mNDVI in our models (as NDVI correlates with net primary productivity), but it is still important to consider possible effects of sampling bias on analyses such as these.

### Conclusion

We have presented the most comprehensive analysis to date of how ecological traits affect bee species' responses to human impacts in Europe. Our results suggest that conservation and management activities should not simply focus on particular land uses or particular traits, but how they interact. Our findings have implications for ecosystem services and food security for two reasons. First, many of the traits affecting species' sensitivities to land use also influence pollination efficiency (de Bello *et al*. 2010). Secondly, trait‐based vulnerability of species also reduces functional diversity (Forrest *et al*. 2015), which is important for insurance against disturbances, pollination efficiency (Albrecht *et al*. 2012) and stability under climate change (Bartomeus *et al*. 2013b). However, to fully understand the implications for pollination provision, further data are required on how traits influence pollination efficacy.

## Data accessibility

The majority of data included in this analysis will be published as part of the PREDICTS database (metadata are already published in Hudson, Newbold *et al*. 2014) and will be hosted by the Natural History Museum's Data Portal (http://data.nhm.ac.uk). Other data are owned by the data collectors: see Table S1.2 for details of all data sources.

## Supporting information


**Appendix S1:** Diversity data set (including details and references for data used in this study).
**Table S1:** Search terms.
**Table S1.2:** Data sources and sample sizes, with references.
**Table S1.3:** Land‐use class and intensity definitions.
**Figure S1.1:** Map of sites used in analysis.
**Appendix S2:** Species traits data set.
**Appendix S2.1:** List of species included in analysis.
**Table S2.1:** Original and coarsened factor levels of species traits.
**Figure S2.1 and S2.2:** Plots showing distribution of traits across families.
**Appendix S3:** Model Checking.
**Table S3.1:** GVIFs for occurrence model.
**Table S3.2 and S3.3:** GVIFs for abundance model before and after model simplification.
**Figure S3.1:** QQ plot of residuals for abundance model.
**Appendix S4:** Model Results.
**Table S4.1:** Coefficient estimates for occurrence model.
**Table S4.2:** Coefficient estimates for abundance model.
**Table S4.3:** Random effect variances of minimum adequate models for occurrence and abundance.
**Appendix S4.1:** Results for interactions between traits and mNDVI.
**Figure S4.1:** Relationship between mNDVI and ITD for occurrence and abundance model.
**Figure S4.2:** Relationship between mNDVI and flight season duration for occurrence model.
