# Supplementary material for: Ecological traits affect the sensitivity of bees to land‐use pressures in European agricultural landscapes
Source: J Appl Ecol. 2015 Sep 23;52(6):1567–77. doi: 10.1111/1365-2664.12524 (PMC4973690; doi:10.1111/1365-2664.12524)
Supplement: Supplementary file 1 — Appendix S1: Diversity data set (including details and references for data used in this study). Table S1: Search terms. Table S1.2: Data sources and sample sizes, with references. Table S1.3: Land‐use class and intensity definitions. Figure S1.1: Map of sites used in analysis. Appendix S2: Species traits data set. Appendix S2.1: List of species included in analysis. Table S2.1: Original and coarsened factor levels of species traits. Figure S2.1 and S2.2: Plots showing distribution of traits across families. Appendix S3: Model Checking. Table S3.1: GVIFs for occurrence model. Table S3.2 and S3.3: GVIFs for abundance model before and after model simplification. Figure S3.1: QQ plot of residuals for abundance model. Appendix S4: Model Results. Table S4.1: Coefficient estimates for occurrence model. Table S4.2: Coefficient estimates for abundance model. Table S4.3: Random effect variances of minimum adequate models for occurrence and abundance. Appendix S4.1: Results for interactions between traits and mNDVI. Figure S4.1: Relationship between mNDVI and ITD for occurrence and abundance model. Figure S4.2: Relationship between mNDVI and flight season duration for occurrence model. [file JPE-52-1567-s001.pdf]

Ecological traits affect the sensitivity of bees to land-use pressures in European agricultural landscapes. Supporting Information

S1 Diversity Dataset

Table S1.1: Terms used to search the Web of Science database for papers potentially containing useful data.

	Web of Knowledge search terms
1	"(arthropod* OR bee* OR pollinat*) AND (abundance OR diversity) AND (agricultur* OR anthropogenic OR land use OR threat)"
2	"pollinat* AND land-use AND diversity"
3	"pollinat* habitat abundance*"
4	"pollinat* threat"
5	"pollinat* agricultur*"

Table S1.2: Data sources and sample sizes

Reference	Study ID	Country	Sampling years	Number of sites	Number of taxa
Darvill, Knight & Goulson (2004)[1]	Darvill2004 transect	United Kingdom	2001 - 2001	17	2
Quaranta *et al.* (2004)[1]	Quaranta2004 Pisa	Italy	2000 - 2000	2	28
Hanley (2005)[1]	Hanley2005 unpublished	United Kingdom	2004 - 2005	6	10
Diekötter *et al.* (2006)[1]	Diekoetter2006 grid	Germany	2001 - 2001	115	2
Marshall, West & Kleijn (2006)[1]	Marshall2006 sweepnet	United Kingdom	2003 - 2003	42	25
	Marshall2006 transect	United Kingdom	2003 - 2003	42	25
Herrmann *et al.* (2007)[1,2]	Herrmann2007 Abundance	Germany	2005 - 2005	13	1
	Herrmann2007 Colonynumber	Germany	2005 - 2005	13	1
Meyer, Gaebele & Steffan-Dewenter (2007)[1]	Meyer2007 2000data	Germany	2000 - 2000	15	7
	Meyer2007 2005data	Germany	2005 - 2005	15	8
Öckinger & Smith (2007)	Ockinger2007 transect	Sweden	2004 - 2004	36	10

Billeter *et al.* (2008)[1], Diekötter, Billeter & Crist (2008)[1] and Le Féon *et al.* (2010)[1]	Greenveins2001 CzechRepublic01	Czech Republic	2001 - 2001	32	33
	Greenveins2001 Germany01	Germany	2001 - 2001	64	69
	Greenveins2001 Switzerland01	Switzerland	2001 - 2001	80	50
	Greenveins2001 Estonia01	Estonia	2001 - 2001	64	26
	Greenveins2001 Belgium01	Belgium	2001 - 2001	64	22
	Greenveins2001 Netherlands01	Netherlands	2001 - 2001	64	9
	Greenveins2001 Germany02	Germany	2002 - 2002	64	127
	Greenveins2001 France02	France	2002 - 2002	48	54
	Greenveins2001 Belgium02	Belgium	2002 - 2002	64	59
	Greenveins2001 Netherlands02	Netherlands	2002 - 2002	64	40

	Greenveins2001 CzechRepublic02	Czech Republic	2002 - 2002	32	80
	Greenveins2001 Estonia02	Estonia	2002 - 2002	64	61
	Greenveins2001 Switzerland02	Switzerland	2002 - 2002	121	92
Franzén & Nilsson (2008)[1]	Franzen2009 transect	Sweden	2005 - 2005	16	77
Goulson, Lye & Darvill (2008)[1]	Goulson2008 mountains	Poland	2006 - 2006	32	22
Kohler *et al.* (2008)[1]	Kohler2008 pantrap	Netherlands	2004 - 2004	5	4
	Kohler2008 sight	Netherlands	2004 - 2004	5	12
	Kohler2008 windowtrap	Netherlands	2004 - 2004	5	6
	Kohler2008 naturereserve	Netherlands	2005 - 2005	4	11
Knight *et al.* (2009)[1,2]	Knight2009 b.pascuorum	United Kingdom	2004 - 2004	7	1
Albrecht *et al.* (2010)	Albrecht2010 sweepnet	Switzerland	2003 - 2004	101	51
	Albrecht2010 transect	Switzerland	2003 - 2004	101	57
Goulson *et al.* (2010)[1]	Goulson2010 nestdensity	United Kingdom	2007 - 2007	14	2
Redpath *et al.* (2010)[1]	Redpath2010 bumblebees	United Kingdom	2008 - 2008	11	5
Bates *et al.* (2011)[1]	Bates2011 multipletraps	United Kingdom	2009 - 2010	24	57
Blake *et al.* (2011)[1]	Blake bumblebee2008	United Kingdom	2008 - 2008	2	6
	Blake bumblebee	United Kingdom	2009 - 2010	4	6
Connop *et al.* (2011)[1,2]	Connop2009 colonies	United Kingdom	2005 - 2005	5	2

Hanley *et al.* (2011)[1]	Hanley2011 bumblebee	United Kingdom	2007 - 2010	34	5
Holzschuh *et al.* (2011)	Holzschuh2011 abundance	Germany	2007 - 2007	67	1
Power & Stout (2011)[1]	Power2011 transect	Ireland	2009 - 2009	20	6
Samnegård, Persson & Smith (2011)[1]	Samnegard2011 pantrap	Sweden	2009 - 2009	9	31
Schüepp *et al.* (2011)[1]	Schuepp2011 hymenoptera	Switzerland	2008 - 2008	30	7
Weiner *et al.* (2011)	Weiner2011 flowervisitorweb	Germany	2007 - 2007	29	49
Hanley (2011)[1]	Hanley2011 Whitchurch	United Kingdom	2011 - 2011	8	17
Mudri-Stojnić *et al.* (2012)[1,2]	Mudri pollinators	Serbia	2011 - 2011	16	19
Osgathorpe, Park & Goulson (2012)[1]	Osgathorpe2012 Hebrides	United Kingdom	2009 - 2009	23	10
	Osgathorpe2012 Somerset	United Kingdom	2010 - 2010	22	10
Verboven, Brys & Hermy (2012)[1]	Verboven2012 bumblebees	Belgium	2009 - 2009	9	4

[1]Data will be made available in Hudson *et al*, in prep
[2]Data are already published within the referenced paper

Table S1.3: Land-use class and intensity definitions as used in Hudson *et al.* (2014)

**Level 1 Land Use**	**Predominant Land Use**	**Minimal use**	**Light use**	**Intense use**
**No evidence of prior destruction of the vegetation**	**Primary forest**	Any disturbances identified are very minor (e.g., a trail or path) or very limited in the scope of their effect (e.g., hunting of a particular species of limited ecological importance).	One or more disturbances of moderate intensity (e.g., selective logging) or breadth of impact (e.g., bushmeat extraction), which are not severe enough to markedly change the nature of the ecosystem. Primary sites in suburban settings are at least Light use.	One or more disturbances that is severe enough to markedly change the nature of the ecosystem; this includes clear-felling of part of the site too recently for much recovery to have occurred. Primary sites in fully urban settings should be classed as Intense use.
	**Primary Non-Forest**	As above	As above	As above
**Recovering after destruction of the vegetation**	**Mature Secondary Vegetation**	As for Primary Vegetation-Minimal use	As for Primary Vegetation-Light use	As for Primary Vegetation-Intense use
	**Intermediate Secondary Vegetation**	As for Primary Vegetation-Minimal use	As for Primary Vegetation-Light use	As for Primary Vegetation-Intense use
	**Young Secondary Vegetation**	As for Primary Vegetation-Minimal use	As for Primary Vegetation-Light use	As for Primary Vegetation-Intense use
	**Secondary Vegetation (indeterminate age)**	As for Primary Vegetation-Minimal use	As for Primary Vegetation-Light use	As for Primary Vegetation-Intense use

**Human use (agricultural)**	**Plantation forest**	Extensively managed or mixed timber, fruit/coffee, oil-palm or rubber plantations in which native understorey and/or other native tree species are tolerated, which are not treated with pesticide or fertiliser, and which have not been recently (< 20 years) clear-felled.	Monoculture fruit/coffee/rubber plantations with limited pesticide input, or mixed species plantations with significant inputs. Monoculture timber plantations of mixed age with no recent (< 20 years) clear-felling. Monoculture oil-palm plantations with no recent (< 20 years) clear-felling.	Monoculture fruit/coffee/rubber plantations with significant pesticide input.Monoculture timber plantations with similarly aged trees or timber/oil-palm plantations with extensive recent (< 20 years) clear-felling.
	**Cropland**	Low-intensity farms, typically with small fields, mixed crops, crop rotation, little or no inorganic fertiliser use, little or no pesticide use, little or no ploughing, little or no irrigation, little or no mechanisation.	Medium intensity farming, typically showing some but not many of the following: large fields, annual ploughing, inorganic fertiliser application, pesticide application, irrigation, no crop rotation, mechanisation, monoculture crop. Organic farms in developed countries often fall within this category, as may high-intensity farming in developing countries.	High-intensity monoculture farming, typically showing many of the following features: large fields, annual ploughing, inorganic fertiliser application, pesticide application, irrigation, mechanisation, no crop rotation.
	**Pasture**	Pasture with minimal input of fertiliser and pesticide, and with low stock density (*not* high enough to cause significant disturbance or to stop regeneration of vegetation).	Pasture either with significant input of fertiliser or pesticide, or with high stock density (high enough to cause significant disturbance or to stop regeneration of vegetation).	Pasture with significant input of fertiliser or pesticide, *and* with high stock density (high enough to cause significant disturbance or to stop regeneration of vegetation).
**Human use (urban)**	**Urban**	Extensive managed green spaces; villages.	Suburban (e.g. gardens), or small managed or unmanaged green spaces in cities.	Fully urban with no significant green spaces.

Figure S1.1: Sites across Europe for which we have bee species occurrence or abundance measurements.

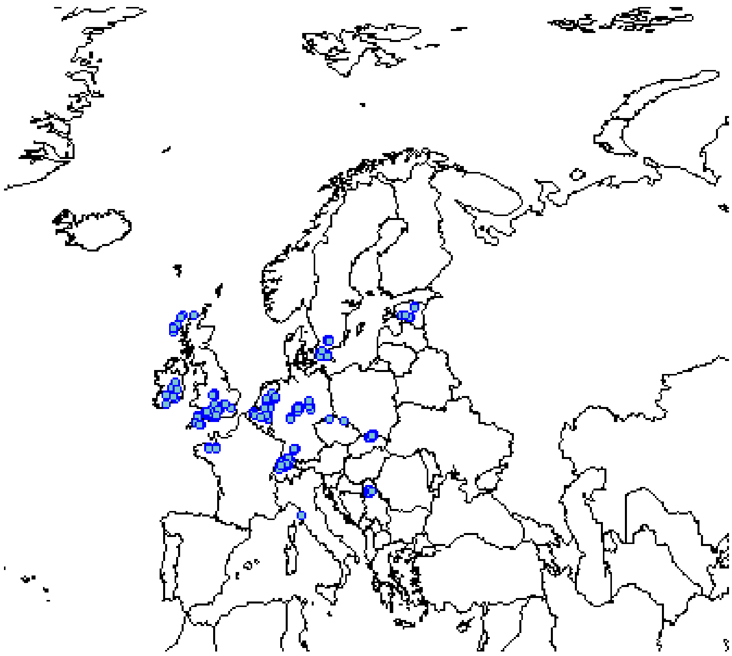

S2 Species traits dataset

S2.1 Species list, based on taxonomy from Michener (2000)

Amegilla albigena

Andrena agilissima

Andrena alfkenella

Andrena angustior

Andrena anthrisci

Andrena barbilabris

Andrena bicolor

Andrena bucephala

Andrena carantonica

Andrena chrysopus

Andrena chrysosceles

Andrena cineraria

Andrena cinerea

Andrena clarkella

Andrena coitana

Andrena combinata

Andrena congruens

Andrena curvungula

Andrena decipiens

Andrena denticulata

Andrena distinguenda

Andrena dorsata

Andrena enslinella

Andrena flavipes

Andrena florea

Andrena floricola

Andrena florivaga

Andrena fucata

Andrena fulva

Andrena fulvata

Andrena fulvida

Andrena fuscipes

Andrena gravida

Andrena haemorrhoa

Andrena hattorfiana

Andrena helvola

Andrena humilis

Andrena labialis

Andrena labiata

Andrena lagopus

Andrena lapponica

Andrena lathyri

Andrena minutula

Andrena minutuloides

Andrena mitis

Andrena nigroaenea

Andrena nigroolivacea

Andrena nigrospina

Andrena nitida

Andrena niveata

Andrena ovatula

Andrena pandellei

Andrena pilipes

Andrena praecox

Andrena proxima

Andrena ruficrus

Andrena semilaevis

Andrena similis

Andrena strohmella

Andrena subopaca

Andrena synadelpha

Andrena tarsata

Andrena tibialis

Andrena trimmerana

Andrena vaga

Andrena varians

Andrena ventralis

Andrena viridescens

Andrena vulpecula

Andrena wilkella

Anthidiellum strigatum

Anthidium manicatum

Anthophora aestivalis

Anthophora dispar

Anthophora furcata

Anthophora plumipes

Anthophora retusa

Apis mellifera

Bombus barbutellus

Bombus bohemicus

Bombus campestris

Bombus cryptarum

Bombus distinguendus

Bombus hortorum

Bombus humilis

Bombus hypnorum

Bombus jonellus

Bombus lapidarius

Bombus lucorum

Bombus magnus

Bombus muscorum

Bombus norvegicus

Bombus pascuorum

Bombus pomorum

Bombus pratorum

Bombus pyrenaeus

Bombus quadricolor

Bombus ruderarius

Bombus ruderatus

Bombus rupestris

Bombus schrencki

Bombus semenoviellus

Bombus soroeensis

Bombus subterraneus

Bombus sylvarum

Bombus sylvestris

Bombus terrestris

Bombus vestalis

Bombus veteranus

Bombus wurflenii

Ceratina cucurbitina

Chelostoma campanularum

Chelostoma distinctum

Chelostoma florisomne

Chelostoma rapunculi

Coelioxys inermis

Coelioxys rufescens

Colletes cunicularius

Colletes daviesanus

Colletes succinctus

Dasypoda hirtipes

Dufourea dentiventris

Eucera eucnemidea

Eucera longicornis

Eucera nigrescens

Halictus confusus

Halictus gemmeus

Halictus maculatus

Halictus rubicundus

Halictus scabiosae

Halictus simplex

Halictus subauratus

Halictus tumulorum

Heriades truncorum

Hoplitis adunca

Hoplitis anthocopoides

Hoplitis claviventris

Hoplitis leucomelana

Hylaeus angustatus

Hylaeus annularis

Hylaeus brevicornis

Hylaeus communis

Hylaeus confusus

Hylaeus difformis

Hylaeus gibbus

Hylaeus gredleri

Hylaeus hyalinatus

Hylaeus nigritus

Hylaeus paulus

Hylaeus punctatus

Hylaeus rinki

Hylaeus signatus

Hylaeus sinuatus

Hylaeus styriacus

Hylaeus variegatus

Lasioglossum albipes

Lasioglossum albocinctum

Lasioglossum brevicorne

Lasioglossum calceatum

Lasioglossum costulatum

Lasioglossum fulvicorne

Lasioglossum glabriusculum

Lasioglossum interruptum

Lasioglossum laevigatum

Lasioglossum laticeps

Lasioglossum lativentre

Lasioglossum leucopus

Lasioglossum leucozonium

Lasioglossum lineare

Lasioglossum lucidulum

Lasioglossum majus

Lasioglossum malachurum

Lasioglossum minutissimum

Lasioglossum minutulum

Lasioglossum morio

Lasioglossum nigripes

Lasioglossum nitidiusculum

Lasioglossum nitidulum

Lasioglossum pallens

Lasioglossum parvulum

Lasioglossum pauxillum

Lasioglossum politum

Lasioglossum puncticolle

Lasioglossum quadrinotatum

Lasioglossum rufitarse

Lasioglossum sexnotatum

Lasioglossum sexstrigatum

Lasioglossum smeathmanellum

Lasioglossum subfasciatum

Lasioglossum villosulum

Lasioglossum xanthopus

Lasioglossum zonulus

Macropis europaea

Macropis fulvipes

Megachile alpicola

Megachile centuncularis

Megachile circumcincta

Megachile ericetorum

Megachile ligniseca

Megachile versicolor

Megachile willughbiella

Melecta albifrons

Melitta haemorrhoidalis

Melitta leporina

Melitta nigricans

Melitta tricincta

Nomada alboguttata

Nomada armata

Nomada bifasciata

Nomada castellana

Nomada fabriciana

Nomada ferruginata

Nomada flava

Nomada flavoguttata

Nomada flavopicta

Nomada fucata

Nomada fulvicornis

Nomada goodeniana

Nomada hirtipes

Nomada lathburiana

Nomada leucophthalma

Nomada marshamella

Nomada panzeri

Nomada ruficornis

Nomada rufipes

Nomada sheppardana

Nomada signata

Nomada striata

Nomada succincta

Nomada zonata

Osmia aurulenta

Osmia bicolor

Osmia bicornis

Osmia brevicornis

Osmia caerulescens

Osmia leaiana

Osmia parietina

Osmia spinulosa

Osmia uncinata

Panurgus banksianus

Panurgus calcaratus

Rhodanthidium septemdentatum

Rophites quinquespinosus

Sphecodes albilabris

Sphecodes crassus

Sphecodes ephippius

Sphecodes ferruginatus

Sphecodes geoffrellus

Sphecodes gibbus

Sphecodes hyalinatus

Sphecodes miniatus

Sphecodes monilicornis

Sphecodes pellucidus

Sphecodes scabricollis

Sphecodes spinulosus

Tetralonia malvae

Trachusa byssina

Table S2.1: Original and coarsened factor levels of species traits

Trait	Coarsened factor levels	Original levels	Rationale
Nesting trait	Excavators	Excavators in the soil or vegetation	This trait was coarsened to represent two distinct nesting strategies: those that build their own holes versus those that don't. Excavators are particular about nesting sites, often requiring hard, bare ground or pithy stems, whilst those that don't excavate use existing cavities or old nesting sites, regardless of nest location.
	Non-excavators	Carder bees, renters, masons, cleptoparasites and social parasites	
Sociality	Obligately solitary	Solitary, solitary or communal, communal, cleptoparasitic	The sociality of the species was defined according to how their offspring are raised, because this relates to reproductive capacity. Social species, or those that raise their young in social nests (such as social parasites), are able to produce greater numbers of offspring because there are more workers to provision those offspring. Primitively eusocial species are able to adjust their reproductive capacity, often according to resource requirements: for example, *Halictus rubicundus* is social in warmer, more resource rich areas but solitary in other areas.
	Not obligately solitary	Highly eusocial, primitively eusocial, solitary/primitively eusocial, polymorphic, social parasites	

Lecty	No Lecty status	No Lecty status	Species with no lecty status are those which do not collect their own pollen, for example cleptoparasites. Phenotypic flexibility can be considered as a form of generalism so species that can be either oligolectic or polylectic are considered in the same category as the pollen generalists.
	Obligately oligolectic	Oligolectic	
	Polylectic/Flexible	Polylectic, oligolectic or polylectic	
Voltinism	Univoltine	Univoltine	Species were split into two categories: those with only one generation per year, and those that do have or can have more than one generation per year, as the latter are predicted to be less impacted by local threats
	Multivoltine/Flexible	Bivoltine, multivoltine, univoltine or bivoltine, univoltine or multivoltine	

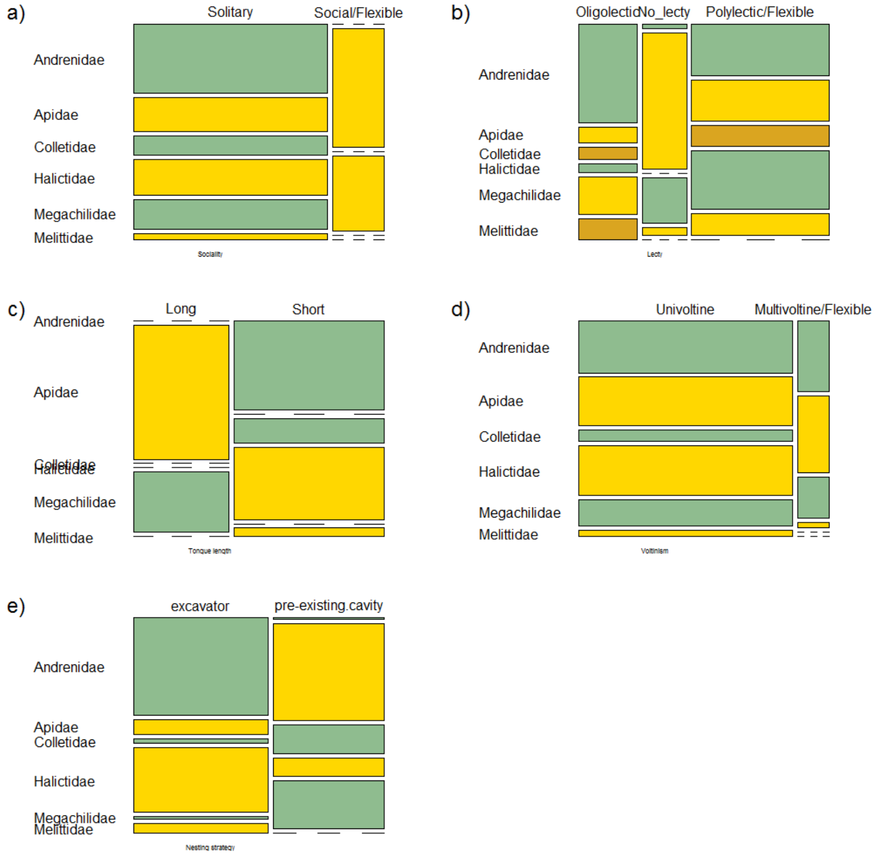

Figure S2.1: Mosaic plots showing the distribution across families of the following categorical traits: a) Sociality, b) Lecty status, c) Tongue length, d) Voltinism, and e) Nesting strategy.

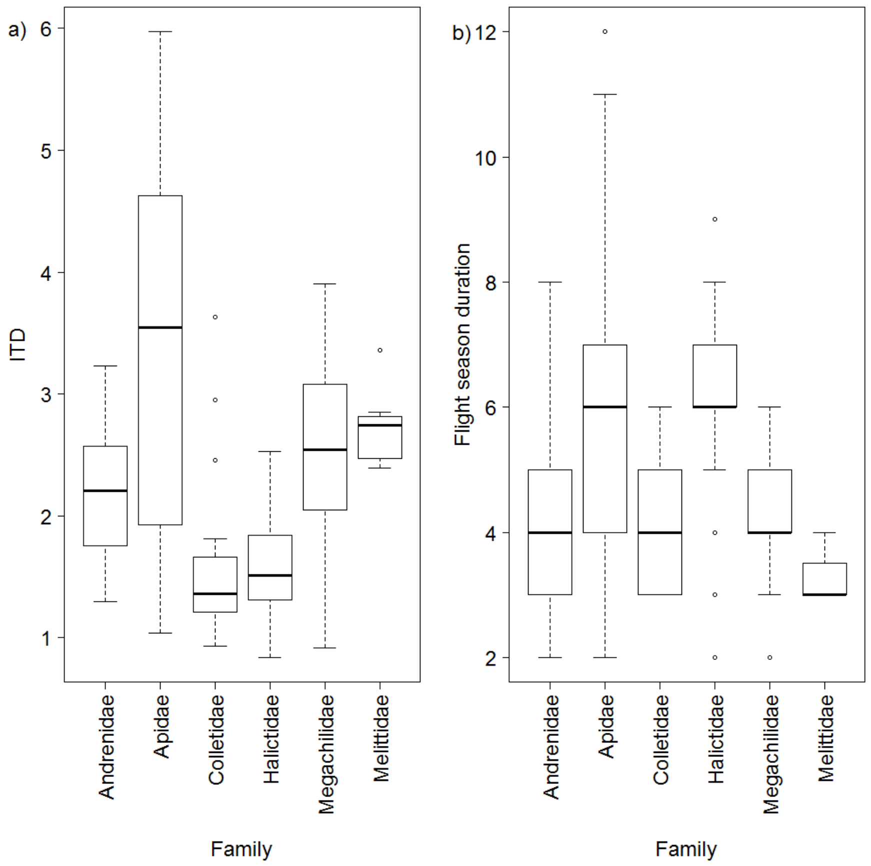

Figure S2.2: Box plots showing the distribution across bee families of a) Inter-tegular distance (ITD) and b) Flight season duration.

S3 Model Checking

Table S3.1: Variance inflation factors (corvif function, Zuur *et al.* 2009) for the dataset used to model probability of species occurrence. GVIF is the generalized variance inflation factor. Df is the degrees of freedom. GVIF scaled by the degrees of freedom gives an indication of how much the standard errors are likely to be inflated due to collinearity between explanatory variables. None of the variables were removed during backwards stepwise model simplification so the GVIFs here are applicable both to the maximal and minimum adequate model for probability of species occurrence.

Explanatory Variable	GVIF	Df	GVIF$^{0.5Df}$
LUI	1.22	5	1.02
mNDVI	1.19	1	1.09
ITD	2.66	1	1.63
Nest construction	4.38	1	2.09
Sociality	1.96	1	1.40
Lecty status	1.85	2	1.17
Voltinism	1.27	1	1.13
Tongue length guild	5.52	1	2.35
Duration of flight season	1.88	1	1.37

Table S3.2: Variance inflation factors (corvif function, Zuur *et al.* 2009) for the dataset used to model abundance of present species, before model simplification. GVIF is the generalized variance inflation factor. Df is the degrees of freedom. GVIF scaled by the degrees of freedom gives an indication of how much the standard errors are likely to be inflated due to collinearity between explanatory variables.

Explanatory Variable	GVIF	Df	$GVIF^{0.5Df}$
LUI	1.33	5	1.03
mNDVI	1.28	1	1.13
ITD	2.98	1	1.73
Nest construction	8.65	1	2.94
Sociality	2.58	1	1.61
Lecty status	1.90	2	1.17
Voltinism	1.60	1	1.26
Tongue length guild	9.60	1	3.10
Duration of flight season	2.83	1	1.68

Table S3.3: Variance inflation factors (corvif function, Zuur *et al.* 2009) for the dataset used to model abundance of present species, after backwards stepwise model simplification based on likelihood ratio tests. GVIF is the generalized variance inflation factor. Df is the degrees of freedom. GVIF scaled by the degrees of freedom gives an indication of how much the standard errors are likely to be inflated due to collinearity between explanatory variables.

Explanatory Variable	GVIF	Df	$\mathrm{GVIF}^{0.5Df}$
LUI	1.31	5	1.03
mNDVI	1.28	1	1.13
ITD	2.96	1	1.72
Sociality	2.58	1	1.61
Lecty status	1.68	2	1.14
Voltinism	1.59	1	1.26
Tongue length guild	3.64	1	1.91
Duration of flight season	2.82	1	1.68

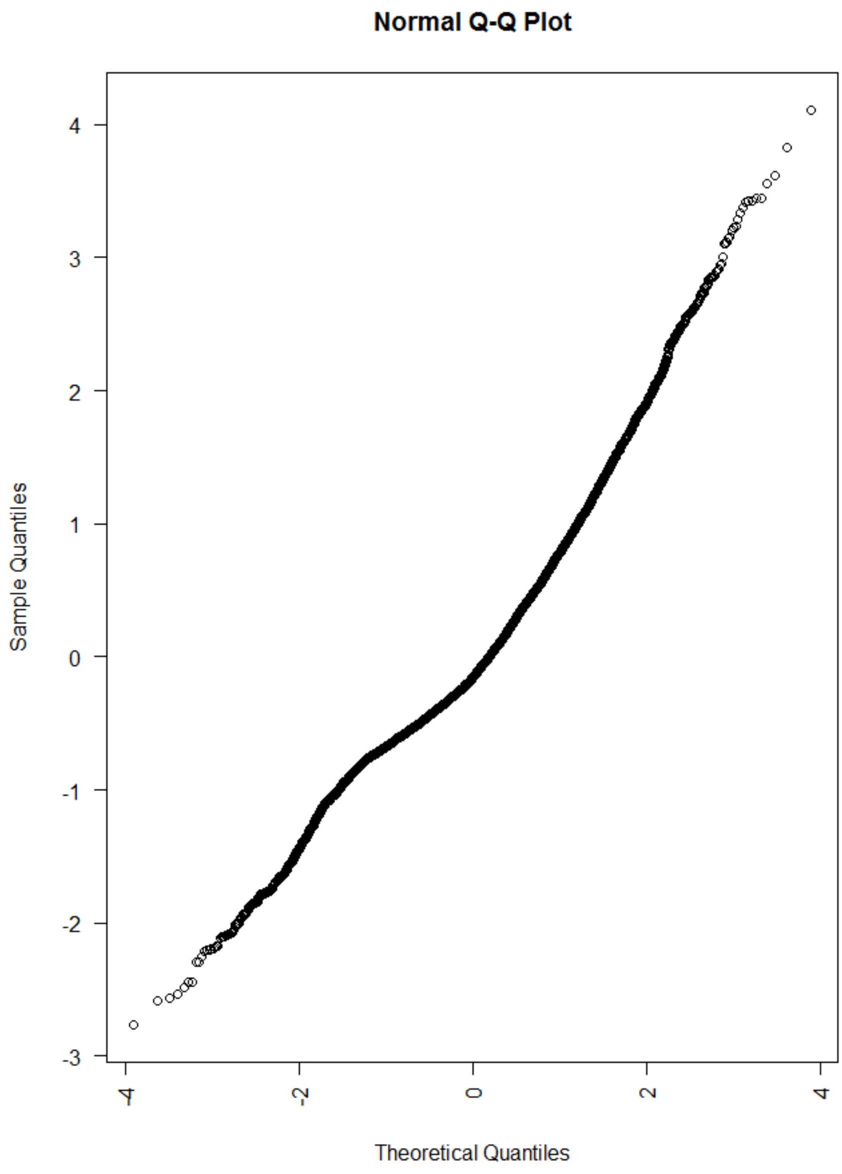

Figure S3.1: Q-Q plot to asssess residuals in the log-transformed abundance model for normality.

S4 Model Results

The following coefficient tables are the model outputs (estimate and standard errors) from mixed effects models in R: A Language and Environment for Statistical Computing version 2.15.3 (R Core Team 2013). These are treatment contrasts, i.e, differences are given between each level and the reference level (oligolectic, solitary, univoltine, short-tongued species in secondary vegetation). The predicted mean of the response variable can be calculated from these tables. For interactions between categorical traits, we can calculate the probability of occurrence of a given trait level in a given land-use class, as a percentage of the probability of occurrence for that same trait level in secondary vegetation. Similarly, this can be done for the abundance of present species. These percentages are provided in the final column of the following coefficients table. Such calculations are not as meaningful for continuous variables, so are not given (denoted by a dash).

Table S4.1: Coefficient estimates (calculated using treatment contrasts in R statistics) and bootstrapped statistics (bias, standard error, confidence intervals) for the model of species occurrence. Significance is assumed if the bootstrapped 95% confidence intervals do not cross zero. The first level of each factor forms part of the intercept terms and so do not explicitly appear in the coefficients table (oligolectic, solitary, univoltine, long-tongued, nest excavating species in secondary vegetation). For each trait level and land-use combination, we also show the probability of species presence as a percentage of the probability of presence for that same trait level in secondary vegetation.

Variable	Coefficient	Original estimate	Standard error	Bias	bootstrapped Standard error	Lower 95% Confidence Interval	Upper 95% Confidence Interval		Difference (%) in Probability of occurrence for trait level in given land use, relative to secondary vegetation
Intercept	(Intercept)	-6.42	1.14	-0.00	0.73	-7.98	-4.51	*	-
LUI	Cropland (Minimal use)	0.02	0.71	-0.01	0.25	-0.49	0.51		2
LUI	Cropland (Light use)	-1.22	0.68	0.01	0.27	-1.79	-0.59	*	-71
LUI	Cropland (Intense use)	-1.81	0.67	0.01	0.20	-2.18	-1.36	*	-84
LUI	Pasture	-1.69	0.73	-0.10	0.54	-2.89	-0.67	*	-82
LUI	Urban	-2.61	1.00	0.02	0.84	-4.23	-0.88	*	-93
mNDVI	mNDVI	7.61	1.21	0.01	0.78	5.96	9.08	*	-
Social Status	Not obligately solitary	-0.61	0.43	-0.05	0.38	-1.57	0.12		-
Lecty Status	No lecty status	2.41	0.77	-0.02	0.50	1.29	3.46	*	-
Lecty Status	Not obligately oligolectic	2.52	0.60	-0.02	0.34	1.93	3.26	*	-
Tongue length	Short tongue	0.82	0.72	-0.04	0.53	-0.45	1.90		-
Voltinism	Not obligately univoltine	0.00	0.28	-0.06	0.25	-0.53	0.58		-
Flight season duration	Flight season duration	0.18	0.09	-0.00	0.08	-0.00	0.34		-
ITD	ITD	0.39	0.16	0.02	0.11	0.18	0.64	*	-
Nest construction	Pre-existing cavity dweller	-0.55	0.49	0.01	0.31	-1.16	0.10		-
LUI × Social Status	Cropland (Minimal use) × Not obligately solitary	-0.44	0.26	0.00	0.15	-0.71	-0.13	*	-34
LUI × Social Status	Cropland (Light use) × Not obligately solitary	-0.65	0.25	0.00	0.13	-0.90	-0.34	*	-85
LUI × Social Status	Cropland (Intense use) × Not obligately solitary	-0.62	0.25	-0.00	0.12	-0.86	-0.36	*	-91
LUI × Social Status	Pasture × Not obligately solitary	0.54	0.33	-0.03	0.35	-0.06	1.25		-68
LUI × Social Status	Urban × Not obligately solitary	0.11	0.34	0.08	0.34	-0.64	0.83		-92
mNDVI × Social Status	mNDVI × Not obligately solitary	1.94	0.47	0.04	0.44	0.80	2.87	*	-
LUI × Lecty Status	Cropland (Minimal use) × No lecty status	0.65	0.47	0.03	0.21	0.19	1.12	*	92

LUI × Lecty Status	Cropland (Light use) × No lecty status	1.05	0.45	0.05	0.20	0.59	1.49	*	-16
LUI × Lecty Status	Cropland (Intense use) × No lecty status	1.26	0.44	0.02	0.17	0.92	1.64	*	-42
LUI × Lecty Status	Pasture × No lecty status	0.06	0.48	0.08	0.34	-0.61	0.83		-80
LUI × Lecty Status	Urban × No lecty status	0.09	0.66	0.09	0.58	-0.99	1.41		-92
LUI × Lecty Status	Cropland (Minimal use) × Not obligately oligolectic	1.07	0.35	0.00	0.14	0.82	1.39	*	187
LUI × Lecty Status	Cropland (Light use) × Not obligately oligolectic	0.62	0.33	0.02	0.13	0.36	0.95	*	-45
LUI × Lecty Status	Cropland (Intense use) × Not obligately oligolectic	0.78	0.32	-0.02	0.11	0.58	1.02	*	-64
LUI × Lecty Status	Pasture × Not obligately oligolectic	0.35	0.35	0.05	0.30	-0.23	1.01		-73
LUI × Lecty Status	Urban × Not obligately oligolectic	0.56	0.57	0.11	0.62	-0.56	2.23		-87
mNDVI × Lecty Status	mNDVI × No lecty status	-4.86	0.92	-0.07	0.56	-6.01	-3.74	*	-
mNDVI × Lecty Status	mNDVI × Not obligately oligolectic	-3.97	0.77	-0.02	0.46	-5.14	-3.06	*	-
LUI × Tongue length	Cropland (Minimal use) × Short tongue	-0.36	0.49	0.03	0.18	-0.68	0.08		-28
LUI × Tongue length	Cropland (Light use) × Short tongue	0.09	0.47	0.02	0.18	-0.30	0.57		-68
LUI × Tongue length	Cropland (Intense use) × Short tongue	0.24	0.46	0.01	0.14	-0.10	0.59		-79
LUI × Tongue length	Pasture × Short tongue	0.58	0.50	0.01	0.30	-0.04	1.19		-67
LUI × Tongue length	Urban × Short tongue	0.09	0.57	-0.01	0.48	-0.82	1.48		-92
mNDVI × Tongue length	mNDVI × Short tongue	-1.72	0.61	0.02	0.41	-2.41	-0.79	*	-
LUI × Voltinism	Cropland (Minimal use) × Not obligately univoltine	0.05	0.21	0.03	0.15	-0.25	0.41		7
LUI × Voltinism	Cropland (Light use) × Not obligately univoltine	-0.01	0.20	0.04	0.12	-0.23	0.33		-71
LUI × Voltinism	Cropland (Intense use) × Not obligately univoltine	0.10	0.20	0.03	0.11	-0.07	0.37		-82
LUI × Voltinism	Pasture × Not obligately univoltine	-1.02	0.24	-0.02	0.19	-1.44	-0.70	*	-93
LUI × Voltinism	Urban × Not obligately univoltine	0.14	0.26	0.04	0.22	-0.19	0.66		-91
LUI × Flight season duration	Cropland (Minimal use) × Flight season duration	0.11	0.05	0.00	0.02	0.05	0.15	*	-
LUI × Flight season duration	Cropland (Light use) × Flight season duration	0.20	0.05	0.00	0.02	0.15	0.26	*	-

LUI × Flight season duration	Cropland (Intense use) × Flight season duration	0.23	0.05	0.01	0.02	0.20	0.27	*	-
LUI × Flight season duration	Pasture × Flight season duration	0.25	0.06	0.00	0.05	0.16	0.36	*	-
LUI × Flight season duration	Urban × Flight season duration	0.23	0.08	-0.01	0.07	0.10	0.37	*	-
mNDVI × Flight season duration	mNDVI × Flight season duration	-0.23	0.10	0.00	0.08	-0.40	-0.07	*	-
LUI × ITD	Cropland (Minimal use) × ITD	-0.15	0.09	0.01	0.04	-0.24	-0.06	*	-
LUI × ITD	Cropland (Light use) × ITD	0.05	0.09	0.01	0.04	-0.03	0.12		-
LUI × ITD	Cropland (Intense use) × ITD	0.11	0.08	0.00	0.04	0.02	0.18	*	-
LUI × ITD	Pasture × ITD	-0.24	0.11	0.02	0.09	-0.41	-0.01	*	-
LUI × ITD	Urban × ITD	0.06	0.12	-0.02	0.09	-0.18	0.20		-
mNDVI × ITD	mNDVI × ITD	-0.67	0.19	-0.00	0.13	-0.96	-0.41	*	-
LUI × Nest construction	Cropland (Minimal use) × Pre-existing cavity dweller	0.77	0.46	0.01	0.17	0.41	1.15	*	121
LUI × Nest construction	Cropland (Light use) × Pre-existing cavity dweller	0.32	0.45	0.01	0.16	-0.05	0.71		-60
LUI × Nest construction	Cropland (Intense use) × Pre-existing cavity dweller	0.07	0.44	0.01	0.14	-0.15	0.46		-82
LUI × Nest construction	Pasture × Pre-existing cavity dweller	0.84	0.48	-0.01	0.34	0.21	1.66	*	-57
LUI × Nest construction	Urban × Pre-existing cavity dweller	1.20	0.54	0.04	0.48	0.35	2.29	*	-76

Table S4.2: Coefficient estimates (calculated using treatment contrasts in R statistics) and bootstrapped statistics (bias, standard error, confidence intervals) for the model of species abundance (when present). Significance is assumed if the bootstrapped 95% confidence intervals do not cross zero. The first level of each factor forms part of the intercept terms and so do not explicitly appear in the coefficients table (Oligolectic, Solitary, Univoltine, long-tongued species in secondary vegetation). For each trait level and land-use combination, we also show the species abundance as a percentage of the abundance for that same trait level in secondary vegetation.

Variable	Coefficient	Original estimate	Standard error	Bias	bootstrapped Standard error	Lower 95% Confidence Interval	Upper 95% Confidence Interval		Difference (%) in abundance for trait level in given land use, relative to secondary vegetation
Intercept	(Intercept)	-1.17	0.63	-0.00	0.62	-2.32	0.04		-
LUI	Cropland (Minimal use)	0.39	0.28	-0.01	0.27	-0.12	0.92		48
LUI	Cropland (Light use)	0.05	0.27	-0.01	0.27	-0.47	0.57		5
LUI	Cropland (Intense use)	-0.06	0.26	-0.01	0.27	-0.59	0.46		-6
LUI	Pasture	-0.21	0.26	-0.01	0.26	-0.75	0.28		-19
LUI	Urban	0.37	0.34	-0.02	0.35	-0.35	1.04		45
mNDVI	mNDVI	3.04	0.72	0.02	0.69	1.64	4.49	*	-
Social Status	Not obligately solitary	0.28	0.18	0.00	0.19	-0.10	0.65		-
Lecty Status	No lecty status	1.15	0.43	0.02	0.45	0.31	2.06	*	-
Lecty Status	Not obligately oligolectic	0.96	0.36	0.00	0.37	0.25	1.69	*	-
Tongue length	Short tongue	0.86	0.37	0.02	0.36	0.17	1.63	*	-
Voltinism	Not obligately univoltine	-0.10	0.18	0.00	0.18	-0.43	0.24		-
Flight season duration	Flight season duration	0.09	0.04	-0.00	0.04	0.02	0.17	*	-
ITD	ITD	0.23	0.08	0.00	0.08	0.06	0.39	*	-
LUI × Social Status	Cropland (Minimal use) × Not obligately solitary	-0.20	0.18	-0.00	0.18	-0.54	0.18		21
LUI × Social Status	Cropland (Light use) × Not obligately solitary	-0.26	0.17	-0.00	0.18	-0.60	0.11		-19
LUI × Social Status	Cropland (Intense use) × Not obligately solitary	-0.27	0.17	-0.00	0.17	-0.61	0.08		-28
LUI × Social Status	Pasture × Not obligately solitary	0.29	0.19	-0.00	0.20	-0.07	0.68		9
LUI × Social Status	Urban × Not obligately solitary	0.02	0.21	-0.01	0.22	-0.42	0.43		48
mNDVI × Lecty Status	mNDVI × No lecty status	-1.92	0.64	-0.02	0.65	-3.22	-0.71	*	-
mNDVI × Lecty Status	mNDVI × Not obligately oligolectic	-1.34	0.53	-0.01	0.54	-2.45	-0.28	*	-
LUI × Tongue length	Cropland (Minimal use) × Short tongue	0.26	0.16	-0.00	0.16	-0.05	0.59		93
LUI × Tongue length	Cropland (Light use) × Short tongue	0.36	0.15	-0.00	0.15	0.06	0.68	*	51

LUI × Tongue length	Cropland (Intense use) × Short tongue	0.35	0.15	-0.00	0.15	0.06	0.67	*	34
LUI × Tongue length	Pasture × Short tongue	0.52	0.16	0.00	0.16	0.22	0.86	*	36
LUI × Tongue length	Urban × Short tongue	0.32	0.18	-0.00	0.18	-0.03	0.67		99
mNDVI × Tongue length	mNDVI × Short tongue	-1.70	0.37	-0.01	0.35	-2.40	-1.03	*	-
LUI × Voltinism	Cropland (Minimal use) × Not obligately univoltine	0.13	0.18	-0.00	0.18	-0.21	0.47		69
LUI × Voltinism	Cropland (Light use) × Not obligately univoltine	0.13	0.17	-0.00	0.17	-0.21	0.45		19
LUI × Voltinism	Cropland (Intense use) × Not obligately univoltine	0.15	0.17	-0.00	0.16	-0.16	0.47		10
LUI × Voltinism	Pasture × Not obligately univoltine	-0.46	0.19	0.00	0.18	-0.81	-0.09	*	-49
LUI × Voltinism	Urban × Not obligately univoltine	-0.00	0.19	0.00	0.19	-0.37	0.37		44
LUI × Flight season duration	Cropland (Minimal use) × Flight season duration	-0.09	0.04	0.00	0.04	-0.16	-0.02	*	-
LUI × Flight season duration	Cropland (Light use) × Flight season duration	-0.04	0.04	0.00	0.04	-0.12	0.03		-
LUI × Flight season duration	Cropland (Intense use) × Flight season duration	-0.03	0.04	0.00	0.04	-0.10	0.04		-
LUI × Flight season duration	Pasture × Flight season duration	-0.01	0.04	0.00	0.04	-0.09	0.07		-
LUI × Flight season duration	Urban × Flight season duration	-0.08	0.05	0.00	0.05	-0.18	0.01		-
mNDVI × ITD	mNDVI × ITD	-0.41	0.12	-0.00	0.11	-0.64	-0.19	*	-

Table S4.3: Random effect variances ± one standard deviation.

Random effect	Occurrence	Abundance
Source ID	2.17 ± 1.47	1.38 ± 1.17
Study within source	0.57 ± 0.75	0.08 ± 0.28
Block in study in source	0.27 ± 0.52	0.01 ± 0.12
Family	0.16 ± 0.40	0.07 ± 0.27
Species within family	0.83 ± 0.91	0.12 ± 0.33

S4.1 Interactions between traits and mDNVI

Species with smaller ITD were significantly more sensitive to areas of low habitat quality (mNDVI) than those with larger ITD (Fig. S4.1). Species with narrow dietary breadths were more sensitive to habitat quality, responding significantly more positively to mNDVI than species that are polylectic (occurrence model estimate = -3.97, bCIs:-6.01,-3.74; abundance model estimate = -1.34, bCIs:-2.45, -0.28) or parasitic (occurrence model estimate = -4.86, bCIs:-5.14, -3.06; abundance model estimate, -1.92, bCIs:-3.22, -0.71). Similarly, long-tongues species were more sensitive to mNDVI than short-tonged species (occurrence model estimate = -1.71, bCIs:-1.44, -0.70; abundance model estimate = -1.70, bCIs:-2.40,-1.03). Social species were also more sensitive to decreasing mNDVI than solitary species (occurrence model estimate = 1.94, bCIs:0.80,2.87)

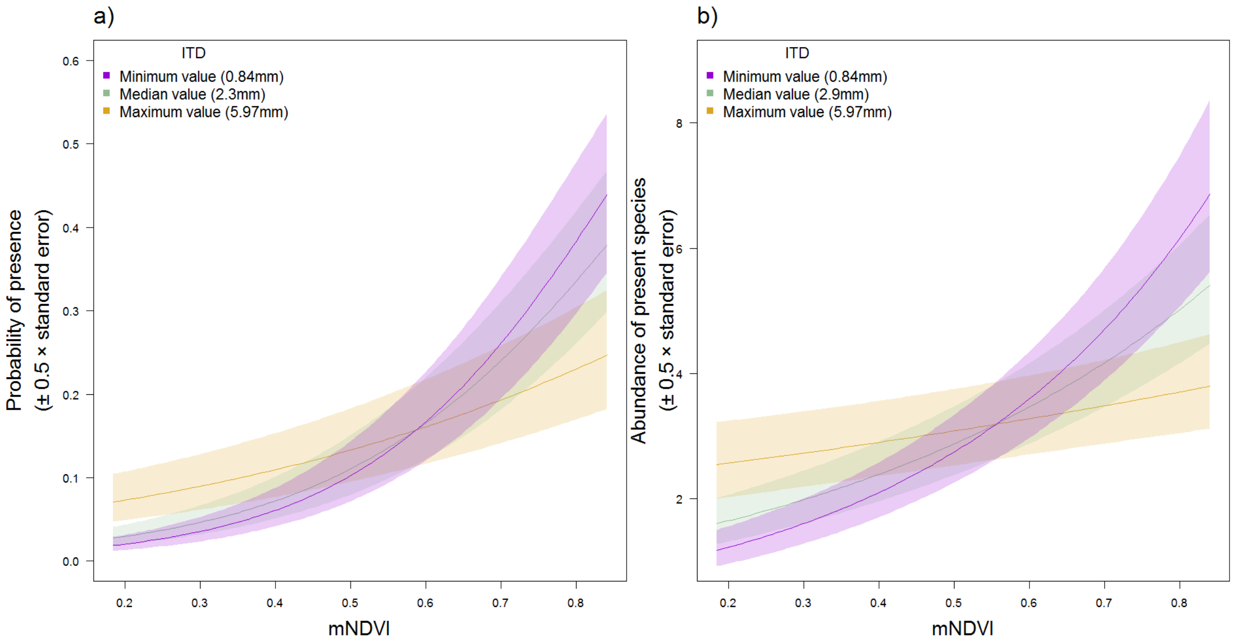

Figure S4.1: Relationship between mNDVI and a) probability of species presence and b) the abundance of present species, predicted for three different body sizes (minimum, median, and maximum ITD values observed in the original dataset). Error bars represent half the standard error (estimated from model coefficients), to ease comparison. The coefficient estimate of ITD × mNDVI was -0.67 (bootstrapped Confidence Intervals, bCIs: -0.96, -0.41) for the occurrence model; and -0.41 (bCIs: -0.64, -0.19) for the abundance model. Where bCIs do not cross zero, the coefficient estimate is taken to be significant.

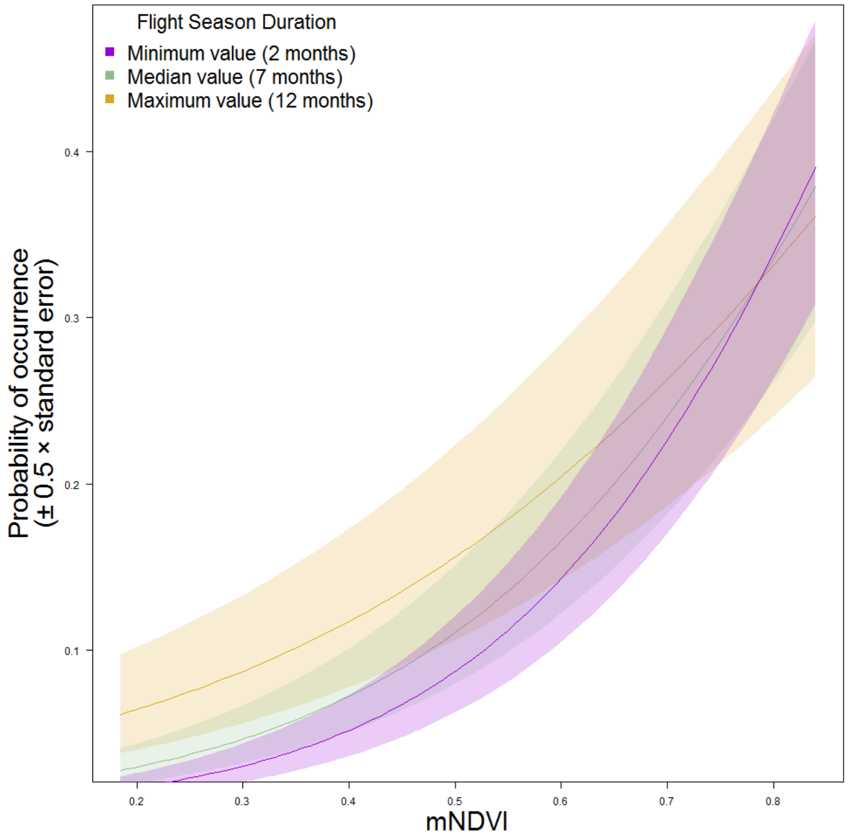

Figure S4.2: Relationship between the probability of species presence and mNDVI, at three different flight season durations (minimum, median, and maximum). Error bars represent half the standard error, to ease comparison between slopes. Coefficient estimate of flight season duration × mNDVI = -0.23 (95% bootstrapped confidence intervals, bCIs = -0.40 and -0.07). Note that where bCIs do not cross zero, the coefficient estimate is taken to be significant.

References

Albrecht, M., Schmid, B., Obrist, M.K., Schüpbach, B., Kleijn, D. & Duelli, P. (2010) Effects of ecological compensation meadows on arthropod diversity in adjacent intensively managed grassland. *Biological Conservation*, **143**, 642–649.

Bates, A.J., Sadler, J.P., Fairbrass, A.J., Falk, S.J., Hale, J.D. & Matthews, T.J. (2011) Changing bee and hoverfly pollinator assemblages along an urban-rural gradient. *PloS one*, **6**, e23459.

Billeter, R., Liira, J., Bailey, D. *et al.* (2008) Indicators for biodiversity in agricultural landscapes: a pan-European study. *Journal of Applied Ecology*, **45**, 141–150.

Blake, R.J., Westbury, D.B., Woodcock, B.A., Sutton, P. & Potts, S.G. (2011) Enhancing habitat to help the plight of the bumblebee. *Pest management science*, **67**, 377–379.

Connop, S., Hill, T., Steer, J. & Shaw, P. (2011) Microsatellite analysis reveals the spatial dynamics of *Bombus humilis* and *Bombus sylvarum*. *Insect Conservation and Diversity*, **4**, 212–221.

Darvill, B., Knight, M.E. & Goulson, D. (2004) Use of genetic markers to quantify bumblebee foraging range and nest density. *Oikos*, **107**, 471–478.

Diekötter, T., Billeter, R. & Crist, T.O. (2008) Effects of landscape connectivity on the spatial distribution of insect diversity in agricultural mosaic landscapes. *Basic and Applied Ecology*, **9**, 298–307.

Diekötter, T., Walther-Hellwig, K., Conradi, M., Suter, M. & Frankl, R. (2006) Effects of landscape elements on the distribution of the rare bumblebee species *Bombus muscorum* in an agricultural landscape. *Biodiversity and Conservation*, **15**, 57–68.

Franzén, M. & Nilsson, S.G. (2008) How can we preserve and restore species richness of pollinating insects on agricultural land? *Ecography*, **31**, 698–708.

Goulson, D., Lepais, O., O'Connor, S., Osborne, J.L., Sanderson, R.A., Cussans, J., Goffe, L. & Darvill, B. (2010) Effects of land use at a landscape scale on bumblebee nest density and survival. *Journal of Applied Ecology*, **47**, 1207–1215.

Goulson, D., Lye, G.C. & Darvill, B. (2008) Diet breadth, coexistence and rarity in bumblebees. *Biodiversity and Conservation*, **17**, 3269–3288.

Hanley, M.E. (2005) Unpublished data of bee diversity in UK croplands and urban habitats.

Hanley, M.E. (2011) Unpublished data of bee diversity in UK croplands and urban habitats.

Hanley, M.E., Franco, M., Dean, C.E. *et al.* (2011) Increased bumblebee abundance along the margins of a mass flowering crop: evidence for pollinator spill-over. *Oikos*, **120**, 1618–1624.

Herrmann, F., Westphal, C., Moritz, R.F.A. & Steffan-Dewenter, I. (2007) Genetic diversity and mass resources promote colony size and forager densities of a social bee (*Bombus pascuorum*) in agricultural landscapes. *Molecular ecology*, **16**, 1167–1178.

Holzschuh, A., Dormann, C.F., Tscharntke, T. & Steffan-Dewenter, I. (2011) Expansion of mass-flowering crops leads to transient pollinator dilution and reduced wild plant pollination. *Proceedings of the Royal Society B: Biological Sciences*, **278**, 3444–3451.

Hudson, L.N., Newbold, T., Contu, S. *et al.* (2014) The PREDICTS database: a global database of how local terrestrial biodiversity responds to human impacts. *Ecology and Evolution*, **4**, 4701–4735.

Knight, M.E., Osborne, J.L., Sanderson, R.A., Hale, R.J., Martin, A.P. & Goulson, D. (2009) Bumblebee nest density and the scale of available forage in arable landscapes. *Insect Conservation and Diversity*, **2**, 116–124.

Kohler, F., Verhulst, J., van Klink, R. & Kleijn, D. (2008) At what spatial scale do high-quality habitats enhance the diversity of forbs and pollinators in intensively farmed landscapes? *Journal of Applied Ecology*, **45**, 753–762.

Le Féon, V., Schermann-Legionnet, A., Delettre, Y., Aviron, S., Billeter, R., Bugter, R., Hendrickx, F. & Burel, F. (2010) Intensification of agriculture, landscape composition and wild bee communities: A large scale study in four European countries. *Agriculture, Ecosystems & Environment*, **137**, 143–150.

Marshall, E.J.P., West, T.M. & Kleijn, D. (2006) Impacts of an agri-environment field margin prescription on the flora and fauna of arable farmland in different landscapes. *Agriculture, Ecosystems & Environment*, **113**, 36–44.

Meyer, B., Gaebele, V. & Steffan-Dewenter, I.D. (2007) Patch size and landscape effects on pollinators and seed set of the Horseshoe vetch, *Hippocrepis comosa*, in an agricultural landscape of Central Europe. *Entomologia Generalis*, **30**, 173–185.

Michener, C.D. (2000) *The Bees of the World.* The John Hopkins University Press, London.

Mudri-Stojnić, S., Andrić, A., Józan, Z. & Vujić, A. (2012) Pollinator diversity (Hymenoptera and Diptera) in semi-natural habitats in Serbia during summer. *Archives of Biological Sciences*, **64**, 777–786.

Öckinger, E. & Smith, H.G. (2007) Semi-natural grasslands as population sources for pollinating insects in agricultural landscapes. *Journal of Applied Ecology*, **44**, 50–59.

Osgathorpe, L.M., Park, K. & Goulson, D. (2012) The use of off-farm habitats by foraging bumblebees in agricultural landscapes: implications for conservation management. *Apidologie*, **43**, 113–127.

Power, E.F. & Stout, J.C. (2011) Organic dairy farming: impacts on insect-flower interaction networks and pollination. *Journal of Applied Ecology*, **48**, 561–569.

Quaranta, M., Ambroselli, S., Barro, P. *et al.* (2004) Wild bees in agroecosystems and semi-natural landscapes. 1997-2000 collection period in Italy. *Bulletin of Insectology*, **57**, 11–61.

R Core Team (2013) *R: A Language and Environment for Statistical Computing.* R Foundation for Statistical Computing, Vienna.

Redpath, N., Osgathorpe, L.M., Park, K. & Goulson, D. (2010) Crofting and bumblebee conservation: The impact of land management practices on bumblebee populations in northwest Scotland. *Biological Conservation*, **143**, 492–500.

Samnegård, U., Persson, A.S. & Smith, H.G. (2011) Gardens benefit bees and enhance pollination in intensively managed farmland. *Biological Conservation*, **144**, 2602–2606.

Schüepp, C., Herrmann, J.D., Herzog, F. & Schmidt-Entling, M.H. (2011) Differential effects of habitat isolation and landscape composition on wasps, bees, and their enemies. *Oecologia*, **165**, 713–721.

Verboven, H.A.F., Brys, R. & Hermy, M. (2012) Sex in the city: Reproductive success of *Digitalis purpurea* in a gradient from urban to rural sites. *Landscape and Urban Planning*, **106**, 158–164.

Weiner, C.N., Werner, M., Linsenmair, K.E. & Blüthgen, N. (2011) Land use intensity in grasslands: Changes in biodiversity, species composition and specialisation in flower visitor networks. *Basic and Applied Ecology*, **12**, 292–299.

Zuur, A.F., Ieno, E.N., Walker, N.J., Saveliev, A.A. & Smith, G.M. (2009) *Mixed effects models and extensions in ecology with R.* No. iii in Statistics for Biology and Health, Springer Science+Business Media, New York.
